# Household food insecurity associated with gestacional and neonatal outcomes: a systematic review

**DOI:** 10.1186/s12884-020-02917-9

**Published:** 2020-04-17

**Authors:** Ana Lucia Pires Augusto, Aléxia Vieira de Abreu Rodrigues, Talita Barbosa Domingos, Rosana Salles-Costa

**Affiliations:** 1 Nutrition Faculty Emília de Jesus Ferreiro, Federal Fluminense University, Mário Santos Braga St., 30, 4th floor, 24020-140 - Niterói, Rio de Janeiro, Brazil; 2 Institute of Nutrition Josué de Castro, Federal University of Rio de Janeiro, Carlos Chagas Filho Av, 373, CCS. 2 andar. Bloco J. 21941-902 - Ilha do Fundão, Rio de Janeiro, Brazil; 3 Institute of Nutrition Josué de Castro, Federal University of Rio de Janeiro, Carlos Chagas Filho Av, 373, CCS. 2 andar. Bloco J. 21941-902 - Ilha do Fundão, Rio de Janeiro, Brazil

## Abstract

**Background:**

Food insecurity (FI) occurs when people lack secure access to sufficient amounts of safe and nutritious food. FI has been associated with negative effects on human health, including during the prenatal and neonatal periods. The objective of this study is to evaluate the consequences of FI for pregnant women’s and newborns’ health.

**Methods:**

A literature search was performed with three independent researchers based on the PRISMA guidelines; the search covered the period of November 2008 to July 2019 and was conducted in the following databases: the US National Library of Medicine at the National Institutes of Health (PubMed), Latin American and Caribbean Health Sciences (LILACS), Cochrane Library, Web of Science, Embase, Scopus and OpenGrey. The terms and descriptors were defined by consulting the Medical Subject Headings (MeSH) and Health Sciences Descriptors (DeCS) platforms and mainly included “food security”, “food insecurity”, “pregnancy” and “newborn”. The studies were selected through a title and abstract review and then a reading of the full text. The quality of the studies and the risk of bias were analysed based on the criteria defined in the “Joanna Briggs Institute Reviewers’ Manual” and by Guyatt and colleagues for interventional studies. The population, study design, FI measurement instruments, FI proportions, outcomes, confounders and results were extracted from the 37 studies that were selected according to the eligibility and quality criteria.

**Results:**

FI ​​proportions ranged from 5.2 to 87%. Most studies were conducted with African populations (42.2%) and applied globally used scales to assess FI (56.7%); 27% of the studies adapted scales. There were wide variations in the instruments used to estimate FI. The main outcomes related to FI included stress, anxiety and depression during pregnancy, followed by dietary quality and dietary diversity. Associations of FI with birth defects, neonatal mortality and the early introduction of animal milk to the infant’s diet were also observed.

**Conclusions:**

It is necessary to pay attention to the diversity of FI measurement instruments before FI results are compared. FI can be a risk factor for depression and stress during pregnancy, as well as for neonatal mortality, newborn health problems and breastfeeding interruption.

**Trial registration:**

This systematic review was registered on PROSPERO (CRD42018109478).

## Background

According to the latest Food and Agricultural Organization (FAO)-World Health Organization (WHO) report in 2018 [1], an increase in the occurrence of severe food insecurity (FI) is evident worldwide, with rates of FI increasing from 8.3% in 2014 to 10.2% in 2017. By definition, FI occurs *“when people lack secure access to sufficient amounts of safe and nutritious food for normal growth and development and an active and healthy life*” [2].

FI has been considered a negative factor affecting the health and well-being of individuals [3–5]. It has been associated with health disorders such as overweight, obesity, high consumption of sugar and low consumption of fruits and vegetables [6].

The effects of FI can especially be observed in more vulnerable groups, such as women. The last report from the FAO in 2019 [7] indicated a higher prevalence of moderate and severe FI among women. Some publications have shown the impact of FI on women of fertile age due to limited access to food [8, 9], which leads to poor dietary intake [9].

In pregnancy, some aspects of a woman’s life may play a major role during the gestational period and for neonatal outcomes, especially in adverse social contexts [10, 11]; such effects were estimated to occur for approximately 70% of pregnant women in Nigeria [12] In this sense, FI can be an important variable for the presence or aggravation of stressful pregnancy events by compromising access to sufficient and quality food, as well as increasing clinical complications during pregnancy and childbirth and in newborns [8, 13, 14]. Some relationships of FI with pregnancy have been documented in previous studies, such as associations of FI with overweight in mothers [5], low birth weight [15], low weight in children, inadequate development, overweight, impaired cognitive development, and behavioural and emotional factors [16–19].

In a narrative literature review, the deleterious associations of FI on pregnant women’s and newborns’ health were reported by Ivers and Cullens [5]. According to the authors, FI was associated with overweight and diabetes among pregnant women and low birth weight and increased vertical HIV transmission in newborns. However, the magnitude of these occurrences on the most frequent health outcomes that affect pregnant women and newborns are unclear.

The search began in the MEDLINE database and the International Prospective Register of Systematic Reviews (PROSPERO) to verify the possible existence of systematic reviews that investigated the relationship of FI with adverse outcomes in pregnancy and childbirth; no publications were found with this purpose in the past 5 years. Therefore, considering the impact of FI on maternal and child health, the aim of the present systematic review was to understand the relationship between FI and adverse clinical-nutritional outcomes for pregnant women and newborns in comparison to those with food security (FS), in an attempt to clarify the “state of the literature” on the theme, trying to verify which outcomes are most frequently associated with FI in these situations, without, however, predefining them.

This systematic review was also justified by the need to verify the methodology used to measure FI in pregnant women’s and newborns’ households among diverse population studies in different countries, therefore this is another objective of this study.

## Methods

The protocol of this systematic review was registered in PROSPERO under the code CRD42018109478 to avoid duplication of the study. Before the study was conducted, a search was performed in the MEDLINE database and the International Prospective Register of Systematic Reviews (PROSPERO) to verify the possible existence of systematic reviews with the same purpose as the present study, and no similar publications were found.

The study was performed following the Preferred Reporting Items for Systematic Reviews and Meta-Analyses (PRISMA) guidelines and the PICO/PECO strategy (which stands for Patient/Population, Intervention/Exposure, Control and Outcome(s). In this review, the “P” was defined as pregnant women and/or newborns; the “E” was defined as food insecurity; the “C” was defined as the group(s) with FS; and the “O” was defined as the outcomes found in the investigation.

First, the search strategies were determined. The databases, terms and descriptors were defined. The terms and descriptors were established through consultation of the Medical Subject Headings (MeSH) and Health Sciences Descriptors (DeCS) platforms to ensure the coverage of the search.

The study selection search was performed in the US National Library of Medicine at the National Institutes of Health (PubMed), Latin American and Caribbean Health Sciences (LILACS), Cochrane Library, Web of Science, Scopus and Embase and OpenGrey (to avoid publication bias) databases using the following terms and descriptors: “food supply”, “household food security”, “household food insecurity”, “food security”, “food insecurity”, “pregnancy”, “infant, newborn”, “food and nutritional security”, “pregnancy” and “newborn”. The search was limited to publications from November 2008 to July 2019. Since no systematic review on FI during pregnancy and the neonatal period was found in this period, the publication date range of the search was set to 10 years and expanded to one more year, to ensure that no relevant study was excluded. The authors considered this period to be adequate based on other systematic reviews. Visser and colleagues [20] investigated FI in vulnerable and malnourished populations and also defined a publication period of the past 10 years for the search.

The steps for the PubMed search were as follows: 1) search “food supply” [MeSH Terms] OR “household food security” [Title/Abstract] OR “household food insecurity” [Title/Abstract] OR “food security” [Title/Abstract] OR “food insecurity” [Title/Abstract]); 2) search “pregnancy” [MeSH Terms]; 3) search “infant”, “newborns” [MeSH Terms]; 4) search the terms from steps 1 AND 2; 5) search the terms from steps 1 AND 3; and 6) search the terms from step 4 OR 5. The same search strategy was used for the other databases, except for LILACS, for which the following search strategy was used: 1) search “household food security” AND “pregnancy” OR “household food security” AND “infant”, “newborns”; 2) search the Portuguese terms “segurançaalimentar e nutricional” [DeCs] AND “Gravidez” [DeCs] OR “segurançaalimentar e nutricional” [DeCs] AND “Recém-nascido” [DeCs].

After the search, all references were imported to a reference manager (EndNote). Articles indexed in more than one database, in other words, duplicates, were identified and removed.

The study selection and evaluation process was divided into three stages, with three evaluators independently reviewing and identifying the relevant studies. At all stages, the inclusion of articles in the study was determined based on the agreement of all three evaluators or at least two evaluators, and disagreements were discussed and resolved by consensus. To assess the agreement among the evaluators, the kappa coefficient was estimated taking into consideration all three evaluators.

First, articles were selected based on the titles and abstracts according to the eligibility criteria. Cross-sectional and longitudinal observational studies and clinical trials were included. Literature review articles, descriptive studies, qualitative methodology articles, articles on women with multiple pregnancies, articles on food safety and articles in which FI was evaluated as the outcome variable were excluded.

Of all the publications that resulted from the search, 840 were excluded because they did not fit the study proposal and therefore did not meet the eligibility criteria. Of the publications selected for full-text review (96), 59 were excluded according to the eligibility criteria. It is important to emphasize that the objective of the present study was to investigate the dimension of food and nutritional security concerned with a lack of access to resources necessary for food acquisition, which constitutes FI. Thus, articles that did not address this dimension were not considered. Although a qualitative methodology enriches discussions about the impact of FI on gestational and neonatal health, qualitative studies were excluded because they did not allow for the quantification of the associations of FI with outcomes in pregnant women and newborns. Some other articles were also excluded, as they were not related to the gestational or neonatal period but instead, investigated the relationship between FI exposure and outcomes in later infancy, in childhood or in relation to adolescent pregnancy. Articles that did not present study results, such as protocols and letters to the editor, were also excluded, since it would be impossible to analyse the FI gestational and neonatal outcomes.

After the selection of articles based on the titles and abstracts, the second step was to read the publications in full, and those that clearly met the inclusion criteria were selected. For the systematization of the article review, a spreadsheet was created where the following items of interest were recorded to avoid selective reporting within studies: article identifying information (i.e., authors, year of publication, country of the study population), FI evaluation method, study design, outcomes, adjustments and confounding variables, and results related to pregnancy and the neonatal period.

During the evaluation of the studies, these items were critically analysed as detailed below. For the FI evaluation method, the evaluators determined whether the instrument was validated (although no study was excluded because of this condition), whether it was a questionnaire/scale or another type of instrument, and whether it had been adapted. For the study design, the study type was determined, and narrative literature reviews as well as qualitative studies were excluded since it would have been impossible to systematize the results. Observational and interventional studies were both included in the review, despite their differences in design, because their outcomes could be analysed separately using the “Joanna Briggs Institute Reviewers’ Manual 2015” [21], which provides guidance for critical quality analysis adapted for both observational and interventional studies (in the appendix). In addition, the criteria proposed by Guyatt and colleagues [22] were also used because they provide additional assistance in the critical assessment of studies.

Regarding outcomes, the researchers analysed all the papers that examined influences on pregnant women’s and newborns’ health, while studies with social outcomes were excluded. In relation to adjustment mechanisms and confounding factors, the evaluators verified whether these elements decreased the quality level of each study. The authors also evaluated whether the results had been obtained without information, selection or specification bias and had been analysed with the correct statistical analyses.

**Fig 1.**
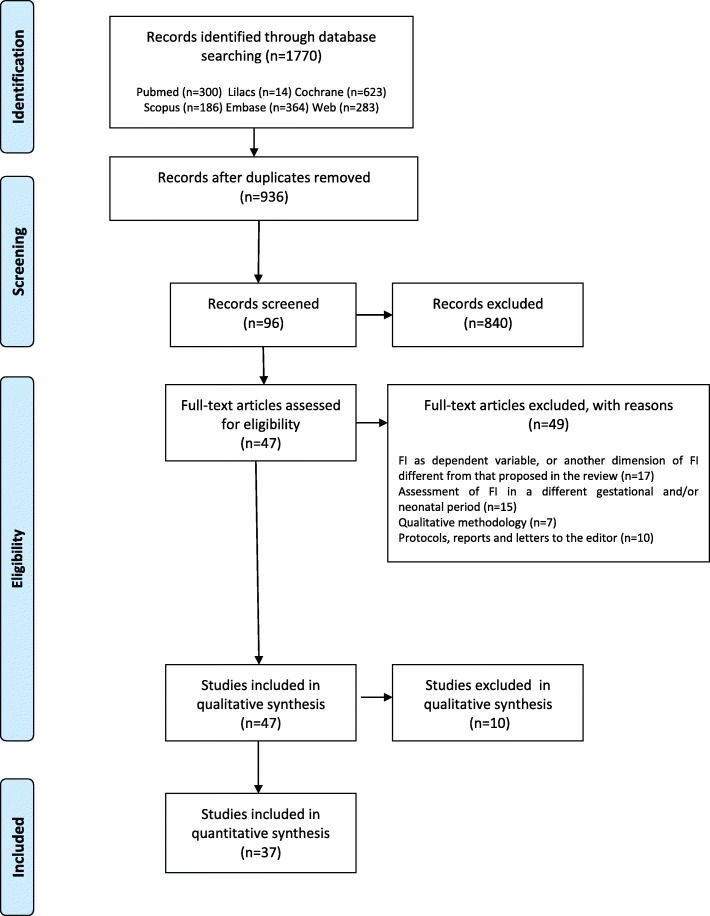
Flow chart of the studies selection. Articles selection flow chart included in this systematic review according to elegibility criteria

An additional critical quality analysis of the studies was carried out using the criteria adopted in the “Joanna Briggs Institute Reviewers’ Manual 2015” [21] and the criteria by Guyatt and colleagues (2008) [22], which allows for the verification of risk of bias in studies. To guarantee the quality of the articles and ensure that the correct articles with valid results were included in the review, a high response rate equal to or greater than 85% of the items was required [23]. The numbers of articles identified and selected from the search and excluded in each step are described in Fig. 1.

## Results

A total of 1770 publications were obtained from the searched databases (*Cochrane = 623, LILACS = 14, PubMed = 300, Scopus = 186, Embase = 364, Web of Science = 283 and OpenGrey = 0*). Of these publications, 834 duplicate papers were excluded; therefore, 936 articles were selected for the first review (titles and abstracts). At this stage, 840 publications were excluded, leaving 66 articles for a full-text review and quality evaluation analysis. After this phase, 59 articles were excluded, and a total of 37 articles were eligible for data extraction (Fig. 1). The kappa coefficient was calculated considering the three evaluators to determine the magnitude of the agreement between them for the eligibility of the studies. The kappa coefficient was 0.79 (95% CI: 0.69; 0.93).

Table S1 presents the 37 articles analysed, showing that the majority (22; 59.5%) of articles were published between 2016 and 2019, ten papers were published between 2012 and 2015, and only four studies were published between 2008 and 2011. An article was also included that was published in 2007 [13] because it provided a counter-reference of interest; this article was obtained from one of the articles that was read [5].

Regarding the study populations, most comprised pregnant women and/or newborns from Africa (16 studies; 43.2%) and the US (11 publications; 29.7%). Seven studies were conducted in Asia (19%), two were conducted in South America (5.4%) and of these, one was conducted in Brazil, and one was conducted in Mexico. One publication (2.7%) was conducted with a US and Puerto Rican population. Regarding the study design, most of the studies used a cross-sectional design (*n* = 20; 54%), followed by a longitudinal design (*n* = 10; 27%), a longitudinal design with intervention or clinical trials (*n* = 4; 10.8%), or a case-control design (*n* = 3; 8.2%). Among the publications analysed, 30 articles (81%) evaluated outcomes associated with FI exclusively during pregnancy, and seven studies (19%) evaluated the relationship between FI and outcomes among newborns [13, 24–29].

Table S1 also shows the methodologies used to assess FI in the studies. In the majority of cases, the Household Food Insecurity Access Scale (HFIAS) from the Food and Nutrition Technical Assistance (FANTA) project of the United States Agency for International Development (USAID) and globally used was the principal questionnaire applied. This scale was used in 15 studies (40.6%), three (8.1%) of which made adjustments to the instrument used to measure FI [30–32] and 12 of which used the original scale. Five studies (13.5%) used the Household Food Security Scale (HFSS) from the United States Department of Agriculture (USDA), an instrument that has been validated for the US population, without any adaptations. Six other publications (16.3%) applied this same scale with adaptations [25, 33–35, 30, 36] and in four articles (10.8%), the authors chose to use the six-item short version of the HFSS [13, 33–35]; however, Carmichael and colleagues [13] used a five-item short version instead of the six-item version, which represents a risk for bias found among the selected studies. The adaptation of instruments without validation procedures may be considered another risk of bias. In one study (2.7%), the HFIAS was applied along with a validated dietary insufficiency question: “How many days did you go hungry last week?“ [36]. In Latin America, some local scales were used; for example, in Brazil, the Brazilian Food Insecurity Scale (EBIA), which has been validated for use in this country, was applied in a study that verified the association between FI and anaemia in adult pregnant women in northeastern Brazil [37]. The Latin American and Caribbean Scale (ELCSA) was used in Mexico to investigate the relationship between FI and hearing disorders at birth. Two studies [38, 39] (5.4%) used the 9-item individually focused food insecurity access scale (IFIAS) that was validated in 2015 in Uganda in a survey of pregnant women [40]. The remaining studies used individual questions to assess FI (Table S1).

Regarding the way to evaluate FI, some studies (*n* = 6, 16.3%) considered FI a continuous variable and used mean scores for analysis [13, 24, 36, 38, 39, 41]. In the others it was considered as a categorical or ordinal. In 12 studies (32.4%), FI was considered an ordinal variable with 4 levels. In four publications, FI was considered an ordinal variable with 3 levels. In 11 studies (29.7%), FI was analysed as a dichotomous variable defined as either FS or FI. Finally, in three publications, FI was treated a dichotomous variable in different ways: one study (2.7%) categorized FI as mild FI versus moderate or severe FI [42]; two studies (5.4%), the adopted dichotomization was food securityFS or mild FI versus moderate or severe FI [37, 43]; and in one study (2.7%), FI was considered either severe or not severe [44].

Table S1 also shows that the proportion of the population with FI found in the studies included in this review was quite diverse, ranging from 5.2% in North Carolina [45] to 87% in South Africa [46]. Some studies also evaluated the proportion of the population with FI severe forms, ranging from 14.2% in Bangladesh [31] to 80% in Uganda [47].

Table S2 presents the studies that evaluated the effect of FI on gestational outcomes. In eleven studies (29.7%), women from families with FI had higher chances of depression and/or anxiety or stressful events in their lives, which was the most prevalent outcome. Among the studies that investigated this association, Natamba and colleagues [38] found that a lack of social support in pregnant women with depressive symptoms was associated with FI. Poor dietary quality and/or dietary diversity and/or inadequate nutrient intake was the second most investigated outcome in eight publications (22.2%); this outcome was associated with FI in six of the publications, but in two publications, the authors found no association [14, 34]. The relationship between the gestational weight gain and/or inadequate nutritional status of pregnant women was investigated, with or without an examination of pregnancy complications and nutritional consumption outcomes, in four studies (the studies published by Laraia and colleagues [9, 45, 48] and by Widen et al. [39]. The association of anaemia with FI was verified in three studies [9, 32, 37]. However, Lebso and colleagues [32] found no associations between anaemia and FI. Other outcomes were investigated as a consequence of FI exposure, such as antiretroviral pharmacokinetics [47], alcohol and/or drug use [35, 46], pregnant women’s beliefs about breastfeeding [43] and quality of life [49]. In all these studies, FI was associated with the outcome surveyed.

The relationship between FI and neonatal health was observed in seven studies (19%). Carmichael and colleagues [13] found that FI during pregnancy was strongly associated with neonatal abnormalities (i.e., transposition of the great vessels at the base of the heart, tetralogy of Fallot, spina bifida and cleft palate). Campbell and colleagues [24] observed an association of FI with neonatal mortality. In the study by Hanselman and colleagues [25], the authors observed that FI reduced the chance of the early introduction of artificial milk-based feeding in newborns by 62%.

Regarding the association of FI and neonatal outcomes, other surveys investigated low birth weight (LBW) [28], prematurity [27], hearing disorders [26] and neonatal abstinence syndrome [29].

## Discussion

This systematic review demonstrated that some researchers show interest in investigating FI gestational outcomes but not neonatal outcomes. Of the 37 studies analysed, only seven aimed assess neonatal outcomes (Table S1). The remaining 30 sought to investigate the relationship between FI and outcomes in pregnant women, highlighting the role of stress, anxiety, depression disorders and poor quality of diet as major factors arising from, or at least closely related to, FI. These findings are particularly worrying, as they increase physical and psychosocial vulnerability in the gestational and neonatal periods [38], including the risk of mortality [24], prematurity [27], and neonatal health problems [13, 26, 29], among others.

Some authors showed how FI may be related to adverse consequences for pregnant women and newborns; for example, de Oliveira and colleagues [37] reported an anaemia prevalence of 28.3% among pregnant women in a northeastern Brazilian town. In this region, anaemia is considered a moderate public health risk. Bartelink and colleagues [47] observed severe FI associated with undernutrition, which reduced serum exposure to antiretroviral drugs in pregnant HIV women in Uganda. According to the study, FI explained 14% of the variability in exposure to the drugs. In a study on newborn mortality, the highest neonatal mortality rate was found among women from households with FI in Indonesia [24]. These findings evidence the importance of the investigation of the adverse associations of FI with outcomes during pregnancy and the neonatal period.

In this review, it was possible to note that most publications were concentrated in the past 3 years, coinciding with the period of rising world hunger, notably in Africa and Latin America, according to the 2018 and 2019 FAO reports [1, 7]. The direct relationship between extreme poverty and adverse social factors, such as low education, poor housing conditions, poor sanitation, and poor access to adequate food and health care among other conditions [50, 51], contributes to the high occurrence of FI on these continents [1], justifying the largest number of studies of FI, including those with pregnant women and newborns.

The impact of FI during the gestational and neonatal periods is particularly worrying due to the impact on quality of life, as Moafi and colleagues [49] demonstrated in their study of pregnant women in which FI was associated with very low quality of life scores due to the physical components of the score. This finding points to the great vulnerability of these periods.

Despite neonatal vulnerability, the present systematic review showed that of the 37 articles selected, only seven (19%) investigated the outcomes associated with FI in this period, such as birth defects [13], birth mortality^25,^ introduction of artificial feeding [25], prematurity [27], LBW [28], hearing disorders [26], and neonatal abstinence syndrome [29].

On the other hand, the number of studies observing the relationship between FI and the health outcomes of pregnant women was much higher (*n* = 30). In other words, although the investigation of FI and health outcomes in pregnant women is a favourable finding of this review, as such research allows increased attention to the risks posed by FI during pregnancy, unless there are rare clinical complications [8, 9], it seems that this attention does not last in the neonatal period. The discrepancy between the number of publications on pregnant women and on newborns points to a lack of continuity in the follow-up of the prenatal FI ​​situation during neonatal life, highlighting a possible lack of comprehensiveness in gestational-neonatal assistance. This fact certainly has adverse consequences for newborns [52], such as the interruption of breastfeeding [25] among others related to stress [33].

Another point that should be highlighted from this review refers to the form of FI measurement observed in the studies. Most of them used the HFIAS in the original form from the FANTA project, which, according to the authors, is an instrument to measure the components of access to adequate food, and the HFSS from the USDA, which is a validated instrument for the North American population.

Twelve studies used the HFIAS, and the authors of three of the studies made modifications [30–32]. Two of the studies [31, 32] did not describe the validation steps of the adaptations performed, which may represent a risk of bias, and in one study [30], the scale was adapted to the local language and validated.

The 18-item HFSS was used in 11 studies, and six of them reported adapting the HFSS to measure FI [24, 29, 42, 46, 53, 54]. Nunnery and colleagues [54] made adaptations to the scale for pregnant participants in their study; their study was the only included study that was concerned with the specificities of the studied population. This is a relevant subject, as pregnancy introduces special biological and psychosocial needs for women that can interfere with their perceptions of hunger and food security, as pointed out by Hoseini and colleagues [55], who also verified that the chances of pregnancy complications were almost 2 times higher in women with FI than in those with FS (Table S1).

The short version of the HFSS was used in four studies [13, 33–35]. In one study [13], one of the questions from the short version of the HFSS was excluded. In these cases, where both scales were adapted without statistical validation, there was a risk of bias. In a systematic review performed to identify and characterize various experience-based household food security scales and synthesize their psychometric properties, the authors concluded that there are a number of structured scales available in the literature to assess household FI. They observed that the use of these scales is still limited due to the appraisal of few aspects of reliability and validity [56].

This finding is corroborated in the evaluation of the study by Na and colleagues [31], who used the HFIAS scale but modified the recall time to evaluate FI. In the original version of the scale, the recall time was 4 weeks, and in the study by Na and colleagues [31] with Bangladeshi women, the authors adapted the use of the scale to assess the last 6 months prior to the interview. In a study in Ethiopia [32], adaptations were also made to the HFIAS for the local context, but further details on the validity of the modifications performed to estimate FI were not provided, which makes it difficult to determine which aspects related to food access actually were investigated. These are reasons why the mentioned studies can be considered to have risk of bias. Campbell and colleagues [24], in turn, adapted a scale constructed by Melgar-Quinonez and colleagues for Bolivia [57] that was already modified from the HFSS. The authors, however, determined that the external validity of the scale was considered adequate. Similarly, for a survey carried out with pregnant women in Pakistan to determine the association between FI and depression, the HFIAS was adapted to the local language and was validated [30]. Another publication that verified the existence of neonatal abstinence syndrome in association with FI in Boston used two questions from the HFSS, but the instrument was validated. In both cases, there was no risk of bias, but a problem still remained: the limited comparison of results from different studies.

The use of different FI questionnaires/scales and adapted instruments that vary from the original versions generates a robust discussion about the comparability of study results being impaired. When the instruments used in various investigations are distinct from each other, even in their original forms, the comparison and discussion of the results obtained regarding FI in different populations could be limited. Another important point to consider in this discussion is changes to the original form of an already validated instrument to assess FI (for example, through the exclusion of items). This approach could lead to difficulty [56] in comparing observed associations between FI and outcomes during pregnancy and the neonatal period. This limitation is based on the possibility of changes to the original structure of the questionnaire/scale, which restricts the ability to guarantee that the FI construct will be evaluated in the same way.

The comparison of the results of these studies that applied country-specific local scales such as the EBIA and the ELCSA might also involve limitations. Some authors used single questions to measure FI [10]. These questions investigate the inability to buy the necessary food because of a lack of money. In a study that verified the associations between FI and postnatal depression, the authors used a single question to measure FI based on the number of months during the last year when the food the family needed could not be purchased [58]. In another research [36], the authors used a validated single-item measure of food insufficiency that asked about the number of days spent hungry during the last week. However, food insufficiency was used here as a *proxy* of FI, which must be considered in this discussion, as the use of a proxy could shift the focus of the study to the perceived lack of resources to buy food. Therefore, the difference between these methods of measuring FI and the use of more complex scales is very large, and it is thought that the comparison between the results obtained from the use of these varying instruments is quite difficult.

Thus, in the quantification or classification of differences between FI levels, it can be a problem to compare the results of different studies in the evaluation of the outcomes in question. There were three publications in which the authors investigated gestational outcomes associated with FI, and although they did not use adapted instruments, they chose to exclude pregnant women did not answer the FI scales from the study [9, 45, 48]. The authors considered this exclusion to be a study limitation, and it could be considered a risk factor for bias. Castillo-Chavez and colleagues [26] carried out a case-control study that verified the association between food security and hearing disorders in premature newborns. The authors find relevant hearing problems related to FI households. They included only newborns with complete data in their records, which could represent a risk of bias. Similarly, a study in Iran [27] found an association between premature birth and FI in pregnancy; according to the authors, the participants who had not completed the research questionnaires were excluded.

Another situation that may represent a risk of bias was noted in the study of Widen and colleagues [39] with HIV pregnant women, which demonstrated associations between FI and adverse body composition changes. In their survey, women who did not know their HIV status were excluded. This situation could create selection bias.

Another point to be highlighted in this systematic review concerns the kind of FI assessment. Most studies (32.4%) treated FI as an ordinal variable with four levels (from food security to severe food insecurity), allowing the categorization of FI at different levels, for example, in the comparison of mild and severe FI. In contrast, in 29.7% of the studies, FI was considered a dichotomous categorical variable based on the presence or absence of FI (59.2%). This approach makes it impossible to identify the most severe levels of FI, as well as to determine the factors associated with FI, often leading to the over-dimensioning of FI and poor specification of the severe issues of poverty and socio-demographic conditions associated with severe FI. Some instruments such as the North American scale, the HFSS and the EBIA already standardize the form of expression of the variable [59], which in some way facilitates comparisons of studies using the same scales.

Gestational outcomes were the most investigated by the studies included in this review (Table S2). The symptoms of depression and/or anxiety and/or stress associated with FI were the most notable (36.7% of the 30 studies that analysed only pregnant women and 29.7% of all the studies). The analysis of these results allows us to verify the importance of minimizing stress factors in pregnant women’s. FI, in turn, was shown to increase chances of depression [30, 36, 42, 44, 53, 58, 60], stress [45, 61] and anxiety [33]. In addition to the mental health complications of pregnant women, symptoms of stress and depression increased the release of hormones such as corticotropin [13] and could lead to clinical complications such as hyperglycaemia and hypertension [45, 55]. It was also observed that FI increased the prevalence of birth defects [13], neonatal mortality [24] and early weaning [25]. In addition, premature births also occurred due to stressful events in pregnancy [62], and FI was associated with premature birth in pregnancy in an Iranian study [27] and with LBW [28].

Outcomes related to consumption and dietary quality were the most investigated after stress and depression-related events [10, 14, 31, 34, 41, 45, 54, 63], showing the authors’ concern with verifying dietary adequacy among pregnant women and whether dietary adequacy is truly associated with FI. Since most of the instruments that were used to assess FI in the studies included in this review relied on psychometric methodologies to measure access to food in sufficient quantity and quality, pregnant women’s perceptions of access to adequate food did not necessarily correspond to the actual quality or fitness of the diet they consumed. From this perspective, among the studies that evaluated the relationship between FI and the adequacy of consumption and dietary diversity, in two of the studies, no such association was verified [14, 34]. In the others, this association was observed, suggesting that pregnant women’s perceived lack of sufficient financial resources is truly related to an insufficient quantity or quality of diet for a good portion of pregnancy. It could be expected that this outcome related to quality of the diet should be more frequent than stressful events in women’s life.

Regarding neonatal outcomes, the results found by the authors (Tables S1 and S2) showed a significant association between FI and these outcomes in only seven studies (19%). Carmichael and colleagues [13] observed that FI in pregnant women was strongly associated with the occurrence of neonatal malformations (transposition of the great vessels of the base of the heart, tetralogy of Fallot, spina bifida and cleft palate). These authors found that increased FI risk as indicated by an increased FI score corresponded to an increase in the frequency of health outcomes, which occurred at rates of 3 to 20%. In this research, it was shown that the association of FI with cleft palate and transposition of the great vessels was modified by a low body mass index among pregnant women and that the association of FI with tetralogy of Fallot was modified by folic acid supplementation. Campbell and colleagues [24], who evaluated the association of FI with neonatal mortality, found a 4.6% proportion of neonatal mortality, and families that reported neonatal mortality had significantly higher FI scores than those who did not (2.9 versus 1.72, *p*-value < 0.01) [24]. Hanselman and colleagues [25] reported that FI reduced the likelihood of the early introduction of artificial milk-based feeding into newborn feeding by 62%. Four other outcomes were found in association with neonatal outcomes: LBW (mothers with FI had about 4 times higher odds of LBWthan mothers with FS) [28], prematurity (it was, among mothers with FI, 2 times higher than among those with FS) [27], hearing disorders (severe FI was a high risk factor for hearing disorders, while FS was a protective factor) [26], and neonatal abstinence syndrome (FI condition were strongly associated to the need of abstinence treatment in the adjusted analyses by maternal depression) [29].

Notably, almost all outcomes found by the authors who studied the association between FI and neonatal outcomes were also related to stressful events in pregnancy. Thus, the findings suggest the possibility of stress and its endocrine-metabolic consequences acting as mediators in a causal relationship [64], even though these studies did not evaluate gestational stress (only the results in the neonate). Similarly, it is possible that the relationship between FI and such neonatal outcomes may occur through stress [62, 64].

The present systematic review provided valuable information about the most critical outcomes associated with FI that are harmful for pregnant women and for newborns, who are extremely dependent on the living conditions of their mothers; these results should be carefully considered by professionals in maternal and child health care and public health policy managers. However, some limitations should be considered. The first concerns the loss of some publications that were not identified in the search because their publication language was not English or Spanish, even though there was no language limitation applied to the search criteria in the present systematic review. The second limitation is that this study did not involve a meta-analysis to calculate summary statistical measurements to estimate the relation between the effect of FI on the outcomes observed during pregnancy and the neonatal period. Nevertheless, the systematic review was fundamental to indicate possible gaps that still exist in the research on this theme. One difficulty in the research is that some studies used FI questionnaires/scales with modifications (for example, the exclusion of some items). This approach can be considered to introduce an important bias that may have modified the internal validity of the original validated instruments. Thus, this systematic review can contribute to reinforcing the use of validated scales for the estimation of FI in studies in different countries.

The results described regarding the associations of FI with the health effects of both pregnant women and newborns can be considered representative of these associations since the results were obtained from carefully selected studies conducted in the last 10 years. The choice of the instruments for quality assessment allowed for careful evaluation, including through the positive scoring of publications with a high response rate of the eligible population (over 85%). Thus, the 37 publications analysed in this systematic review provide robust results about FI in the gestational and neonatal periods.

## Conclusions

The results mainly indicate the importance of ensuring the mental health of pregnant women living with FI and paying attention to social factors that can lead to FI to prevent mental disorders such as depression, anxiety and stress, as pregnancy and child birth can themselves lead to biological and psychosocial vulnerabilities. Inadequate nutrient consumption seems to also be an important outcome related to FI in pregnant women according to the present review. Both inadequate nutrient consumption and FI may compromise the development of newborns and the care given to them.

The results of this review demonstrated a high prevalence of FI in women and newborns living in socioeconomically disadvantaged geographical regions, indicating the additional difficulties for this population.

Another important point this study highlighted was the diversity of instruments used to assess FI that can sometimes make comparisons difficult. In addition, the absence of psychometric studies that corroborate the adaptations made to FI instruments compromises the reliability of the results presented by the authors on how to measure FI in different populations.

Additionally, based on the discussion presented, it is suggested that FI is a risk factor for neonatal mortality and some birth defects and disorders in the neonatal period, as well as breastfeeding interruption, low birth weight and prematurity.

In conclusion, given the importance of the increased prevalence of FI worldwide, we hope the present systematic review may prompt additional studies on the relationship between FI and health during pregnancy and the impact of FI on newborns.

## Supplementary information

**Additional file 1:Table S1.** General characteristics from the publications about the relationship between food insecurity (FI), prenatal and neonatal outcomes, potential confoundings, proportion of FI and results identified in the present review. Table referent to all syudies included in this systematic review

**Additional file 2:Table S2.** Proportion (%) and number (n) of studies of clinical or nutritional outcomes observed in pregnant women and newborns associated with food insecurity, identified in the present review Table referent to the proportions of outcomes investigated in this systematic review.

**Additional file 3.** Quality Assessment. The Joanna Briggs Institute Reviewers’ Manual 2015 of quality assessment. Instrument from the Joanna Briggs Institute to assess the quality of the studies included in this systematic review.

## Data Availability

The datasets analysed during the current study are available in its references.

## Abbreviations

FAO: Food and Agricultural Organization
WHO: World Health Organization
FI: Food insecurity
PROSPERO: International Prospective Register of Systematic Reviews
MESH: Medical Subject Headings
DeCS: Health Sciences Descriptors
LILACS: Latin American and Caribbean Health Sciences
HFSS: Household Food Security Scale
USDA: United States Department of Agriculture
HFIAS: Household Food Insecurity Access Scale
EBIA: Brazilian Food Insecurity Scale
IFIAS: Individually Focused Food Insecurity Access Scale
HIV: Human Immune DeficiencyVirus
FS: Food security
BMI: Body mass Index
MAC: MuscularArm Circumference
FANTA: Food and Nutrition Techinical Assistance
USAID: United States Agency for International Development
NTD: Neural Tube Defects

## References

[1] Food and Agriculture Organization of the United States (FAO)/World Health Organization (WHO) (2018). The state of food security and nutrition in the world. Building climate resilience for food security and nutrition.

[2] Food and Agriculture Organization of the United States (FAO)/World Health Organization (WHO) (2009). The state of food insecurity in the world. Economic crises – impacts and lessons learned.

[3] Struble MB, Aomari LL (2003). Position of the American dietetic association: addressing world hunger, malnutrition, and food insecurity. J Am Diet Assoc.

[4] Seligman HK, Laraia BA, Kushel MB (2010). Food insecurity is associated with chronic disease among low-income NHANES participants. J Nutr.

[5] Ivers LC, Cullen KA (2011). Food insecurity: special considerations for women. Am J ClinNutr.

[6] Yang TC, Sahota P, Pickett KE, Bryant M (2018). Association of food security status with overweight and dietary intake: exploration of white British and Pakistani-origin families in the born in Bradford cohort. Nutr J.

[7] Food and Agriculture Organization of the United States (FAO)/World Health Organization (WHO) (2019). The state of food security and nutrition in the world. Safeguardingagainsteconomicslowdownsanddownturns.

[8] Laraia BA, Siega-Riz AM, Gundersen C, Dole N (2006). Psychosocial factors and socioeconomic indicators are associated with household food insecurity among pregnant women. J Nutr.

[9] Laraia BA, Siega-Riz AM, Gundersen C, Dole N (2010). Household food insecurity is associated with self-reported pregravid weight status, gestational weight gain, and pregnancy complications. J Am Diet Assoc.

[10] Brunst KJ, Wright RO, DiGioia K, Enlow MB (2014). Racial/ethnic and sociodemographic factors associated withmicronutrient intakes and inadequacies among pregnant womenin an urban US population. Public Health Nutr.

[11] Borders AEB, Wolfe K, Qadir S, Kim K-Y (2015). Racial/ethnic differences in self-reported and biologic measures of chronic stress in pregnancy. J Perinatol.

[12] Nnakwe NE (2017). The Prevalence of Food Insecurity Among Pregnant Women in a Community-based Intervention Program. FASEB J.

[13] Carmichael SL, Yang W, Herring A, Abrams B, Shaw GM (2007). Maternal food insecurity is associated with increased risk of certain birth defects. J Nutr.

[14] Gamba R, Leung CW, Guendelman S, Lahiff M (2016). Household food insecurity is not associated with overall diet quality among pregnant women in NHANES 1999–2008. Matern Child Health J.

[15] Chowdhury MRK, Khan MMH, Islam R, Perera NKP (2016). Low maternal education and socio-economic status were associated with household food insecurity in children under five with diarrhoea in Bangladesh. ActaPædiatrica.

[16] Bronte-Tinkew J, Zaslow M, Capps R, Horowitz A (2007). Food insecurity works through depression, parenting, and infant feeding to influence overweight and health in toddlers. J Nutr.

[17] Zaslow M, Bronte-Tinkew J, Capps R, Horowitz A (2009). Food security during infancy: implications for attachment and mental proficiency in toddlerhood. Matern Child Health J.

[18] Kral TVE, Chittams J, Moore RH. Relationship between food insecurity, child weight status, and parent-reported child eating and snacking behaviors. J Spec Pediatr Nurs. 2017. 10.1111/jspn.12177. 10.1111/jspn.12177 PMC5398923 28321980

[19] Perez-Escamilla R, Vianna RPT. Food Insecurity and the Behavioral and Intellectual Development of Children: A Review of the Evidence. J Appl Res Children: Informing Pol Children Risk. 2012; http://digitalcommons.library.tmc.edu/childrenatrisk/vol3/iss1/9. Acessed 15 Aug 2019.

[20] Visser J, McLachlan MH, Maayan N (2018). Community-based supplementary feeding for food insecure, vulnerable and malnourished populations - an overview of systematic reviews. Cochrane Database Syst Rev.

[21] The Joanna Briggs Institute (2015). The Joanna Briggs Institute Reviewers’ Manual 2015 Methodology for JBI scoping reviews.

[22] Guyatt G (2008). Rating quality of evidence and strength of recommendation GRADE: an emerging consensus on rating quality of evidence and strength of recommendations. Br Med J.

[23] Rothman KJ (2012). Epidemiology: an introduction. 2nd ed, Oxford.

[24] Campbell AA, de Pee S, Sun K, Kraemer K (2009). Relationship of household food insecurity to neonatal, infant and under-five child mortality among families in rural Indonesia. Food Nutr Bull.

[25] Hanselman B, Ambikapathi R, Mduma E, Svensen E (2018). Associations of land, cattle and food security with infant feeding practices among a rural population living in Manyara. Tanzania BMC Public Health.

[26] Castillo Chávez AM, Torres RM, González VHH (2019). Association between food insecurity and perinatal risk factors with hearing problems in preterm birth. Nutr Hosp.

[27] Dolatian M, Sharifi N, Mahmoodi Z (2018). Relationship of socioeconomic status, psychosocial factors and food insecurity with preterm labor: A longitudinal study. Int J Reprod BioMed.

[28] Gizaw B, Gebremedhin S (2018). Factors associated with low birthweight in North Shewa zone, Central Ethiopia: case-control study. Italian J Pediatrics.

[29] Rose-Jacobs R, Trevino-Talbot M, Lloyd-Travaglini C (2018). Could prenatal food insecurity influence neonatal abstinence syndrome severity?. Addiction..

[30] Ayyub H, Khizran MS, Salam F (2018). Association of antenatal depression and household food insecurity among pregnant women: a crosssectional study from slums of Lahore. J Ayub Med yub Med Coll Abbottabad.

[31] Na M, Mehra S, Christian P, Ali H (2016). Maternal dietary diversity decreases with household food insecurity in rural Bangladesh: a longitudinal analysis. J Nutr.

[32] Lebso M, Anato A, Loha E. Prevalence of anemia and associated factors among pregnant women in Southern Ethiopia: A community based cross-sectional study. PLoS ONE. 2017; 10.1371. 10.1371/journal.pone.0188783 PMC5724831 29228009

[33] Heyningen T, Honikman S, Myer L, Onah MN (2017). Prevalence and predictors of anxiety disorders amongst low income pregnant women in urban South Africa: a cross sectional study. Arch WomensMent Health.

[34] Miller T. A multicenter study of diet quality on birth weight and gestational age in infants of HIV-infected women. MaternChild Nutr. 2017. 10.1111/mcn.12378. 10.1111/mcn.12378 PMC5575978 27863014

[35] Onah MN, Field S, Heyningen T (2016). Predictors of alcohol and other drug use among pregnant women in a peri-urban south African setting. Int J Ment Health Syst.

[36] Tsai AC, Tomlinson M, Comulada WS, Rotheram-Borus MJ (2016). Food insufficiency, depression, and the modifying role of social support: evidence from a population-based, prospective cohort of pregnant women in peri-urban South Africa. SocSci Med.

[37] de Oliveira ACM, Barros AMR, Ferreira RC (2015). Risk factors associated among anemia in pregnancy women of network public health of a capital of Brazil northeastern. RevBrasGinecol Obstet.

[38] Natamba BK, Mehta S, Achan J, Stoltzfus RJ, et al. The association between food insecurity and depressive symptoms severity among pregnant women differs on social support category: a cross-sectional study. Maternal Child Nutrition. 2017. 10.1111/mcn.12351. 10.1111/mcn.12351 PMC6865987 27507230

[39] Widen EM, Tsai I, Collins SM, et al. HIV infection and increased food insecurity are associated with adverse body composition changes among pregnant and lactating Kenyan women. Eur J Clin Nutr. 2018. 10.1038/s41430-018-0285-9. 10.1038/s41430-018-0285-9 PMC6668710 30185898

[40] Natamba BK, Kilama H, Arbach A, Achan J (2015). Reliability and validity of an individually focused food insecurity access scale for assessing inadequate access to food among pregnant Ugandan women of mixed HIV status. Public Health Nutr.

[41] Gebremedhin S, Enquselassie F, Umeta M (2011). Prevalence of prenatal zinc deficiency and its association with socio-demographic, dietary and health care related factors in rural Sidama, Southern Ethiopia: a cross-sectional study. BMC Public Health.

[42] Sidebottom AC, Hellerstedt WL, Harrison PA, Hennrikus D (2014). An examination of prenatal and postpartum depressive symptoms among women served by urban community health centers. Arch Womens Ment Health.

[43] Webb-Girard A, Cherobon A, Mbugua S, Kamau-Mbuthia E (2012). Food insecurity is associated with attitudes towards exclusive breastfeeding among women in urban Kenya. Maternal andChildNutrition.

[44] Garman EC, Schneider M, Lund C (2019). Perinatal depressive symptoms amonglow-income South African women at risk of depression: trajectories and predictors. BMC Pregnancy Childbirth..

[45] Laraia B, Vinikoor-Imler LC, Siega-Riz AM (2015). Food insecurity during pregnancy leads to stress, disordered eating, and greater postpartum weight among overweight women. Obesity..

[46] Eaton LA, Pitpitan EV, Kalichman SC, Sikkema KJ (2014). Food insecurity and alcohol use among pregnant women at alcohol serving establishments in South Africa. Prev Sci.

[47] Bartelink IH, Savic RM, Mwesigwa J, Achan J (2014). Pharmacokinetics of lopinavir/ritonavir and efavirenz in food insecure HIV-infected pregnant and breastfeeding women in Tororo, Uganda. J ClinPharmacol.

[48] Laraia B, Epel E, Siega-Riz AM (2013). Food insecurity with past experience of restrained eating is a recipe for increased gestational weight gain. Appetite..

[49] Moafi F, Kazemi F, Siboni FS, Alimoradi Z (2018). The relationship between food security and quality of life among pregnant women. BMC Pregnancy Childbirth.

[50] Panigassi G, Segall-corrêa AM, Marin-Leon L, Pérez-Escamilla R (2008). Food insecurity as an indicator of inequity:analysis of a population survey. Cad SaudePública.

[51] Jones AD, Ngure FM, Pelto G, Young SL (2013). What are we assessing when we measure food security? A compendium and review of current metrics. Adv. Nutr..

[52] Cook JT, Frank DA, Berkowitz C, Black MM (2004). Food insecurity is associated with adverse health outcomes among human infants and toddlers. J Nutr.

[53] Hromi-Fiedler A, Bermúdez-Millán A, Segura-Pérez S, Pérez-Escamilla R (2011). Household food insecurity is associated with depressive symptoms among low-income pregnant Latinas. Matern Child Nutr.

[54] Nunnery DL, Labban JD, Dharod JM (2017). Interrelationship between food security status, home availability of variety of fruits and vegetables and their dietary intake among low-income pregnant women. Public Health Nutr.

[55] Hoseini KS, Kazemil F, Alimoradi Z (2018). Association between household food security and pregnancy. Social Health Behav.

[56] Marques ES, Reichenheim ME, Moraes CL, Antunes MML, Salles-Costa R (2014). Household food insecurity: a systematic review of the measuring instruments used in epidemiological studies. Public Health Nutr.

[57] Melgar-Quinonez HR, Zubieta AC, MkNelly B, Nteziyaremye A (2006). Household food insecurity and food expenditure in Bolivia, Burkina Faso, and the Philippines. J Nutr.

[58] Murray L, Dunne MP, Vo TV, Anh PNT (2015). Postnatal depressive symptoms amongst women in Central Vietnam: a cross-sectional study investigating prevalence and associations with social, cultural and infant factors. BMC Pregnancy Childbirth.

[59] Pérez-Escamilla R, Segall-Corrêa AM (2008). Food insecurity measurement and indicators. Rev Nutrs Camp.

[60] Woldetensay YK, Belachew T, Biesalski HK (2018). The role of nutrition, intimate partner violence and social support in prenatal depressive symptoms in rural Ethiopia: community based birth cohort study. BMC Pregnancy Childbirth..

[61] Jebena MG, Taha M, Nakajima M, Lemieux A (2015). Household food insecurity and mental distress among pregnant women in southwestern Ethiopia: a cross sectional study design. BMC Pregnancy Childbirth.

[62] Cole-Lewis HJ, Kershaw TS, Earnshaw VA, Yonkers KA (2014). Pregnancy-specific stress, preterm birth, and gestational age among high-risk young women. Health Psychol.

[63] Kang Y, Hurley KM, Ruel-Bergeron J, et al. Household food insecurity is associated with low dietary diversity among pregnant and lactating women in rural Malawi. Public Health Nutr. 2018:1–9. 10.1017/S1368980018002719 PMC10260502 30378520

[64] Coussons-Read ME, Lobel M, Carey JC, Kreither MO (2012). The occurrence of preterm delivery is linked to pregnancy-specific distress and elevated inflammatory markers across gestation. Brain Behavior Immunity.

