# Supplementary material for: Household food insecurity associated with gestacional and neonatal outcomes: a systematic review
Source: BMC Pregnancy Childbirth. 2020 Apr 17;20:229. doi: 10.1186/s12884-020-02917-9 (PMC7164154; doi:10.1186/s12884-020-02917-9)
Supplement: Supplementary file 1 — Additional file 1:Table S1. General characteristics from the publications about the relationship between food insecurity (FI), prenatal and neonatal outcomes, potential confoundings, proportion of FI and results identified in the present review. Table referent to all syudies included in this systematic review [file 12884_2020_2917_MOESM1_ESM.doc]

Table 1: General characteristics from the publications about the relationship between food insecurity (FI), prenatal and neonatal outcomes, potential confoundings, proportion of FI and results identified in the present review.

| Authors, year of publication, country | Population | Study design | FI evaluation | FI variable presentation | Outcomes | Potential counfoundings | Propor-tions  of FI | Results |
| --- | --- | --- | --- | --- | --- | --- | --- | --- |
| Ayyub et al., 2018 (Pakistan) | 367 pregnant women from Lahore (Pakistan)  OBS: sample size properly estimated | Cross-sectional study to assess the association between food insecurity and depression | Adapted and validated Household Food Insecurity Access Scale – Food and Nutrition Techinical Assistance from the United States Agency for International Development (HFIAS/FANTA/  USAID)*** | FI as ordinal variable  (4 levels of FI) | Depression | Maternal age, unwanted pregnancy, death of close relative within 1 year and intimate partner violence | 46% of FI | 53.3% of depression in FI women and 27.8%in FS women. FI women were 2.5 times more prone to antenatal depression than those FS (AOR^#^= 2.58, 95%CI^##^: 1.64–4.075) |
| Bartelink  et al., 2014  (USA) | 221 HIV Pregnant women  (Uganda) | Randomized Clinical Trial to assess the serum exposition to antiretroviral drugs in association to FI in pregnant women | HFIAS/FANTA/  USAID | FI as ordinal variable  (4 levels of FI) | Pharmaco-kinetics of  lopinavir/ritonavir and efavirenz | Ethnicity, Body Mass Indez (BMI), muscular arm circumference (MAC), gestational weight gain, bioavilabity and oral drug clearance | 80% of severe FI | Severe FI was associated to undernutrition that reduced the serum exposition to the antiretrovirals drugs. |
| Brunst et al., 2014  (USA) | 274 Pregnant women (Boston/  USA) | Transversal study with data of a prospective cohort to investigate the associations between sociodemographic characteristics and stress factors including FI and micronutrients/antioxidants consumption | Two items assessed economic-related FI from two diferent scales* | FI was  evaluated considering  two levels  (food security versus FI) | Micro-nutrients/  antioxidants consumption | Pre pregnancy BMI, women age and tobacco use | 27.8% of FI | FI was associated with inadequated consumption of iron, betaine, thiamin and niacin.  Women wiht high consumption of magnesium (35%) had lower frequncy of FI (p-value<0,01). There was no association between the acid folic consumption and FI. |
| Campbel et al., 2009  (USA) | Newborn and children from 26,339 households  from Indonesia | Cross-sectional investigation of neonatal and under-five mortality associated to FI exposure | Adaptation of a proposed scale for populations in Bolivia, Burkina Faso, and the Philippines based on US HFSS Module** | FI as continous  variable  considering the sum  of positive response for each item (0 to 9) | Neonatal and under-5 mortality | Maternal age, education, BMI, smoking, number of household residents, monetary income from weekly spending, use of iodized salt | - | Food insecurity score was related to neonatal mortality after adjusted by confoundings variables (OR=0.05 (95%CI 1.02-1.09; p-value=0.003) |
| Castillo Chávez, et al., 2019  (MEXICO) | 140 premature newborns from 30 to 37 weeks of gestation (35 cases and 105 controls) in Guanajuaro (México) | Case-control study to analyze the association between food security and prenatal risk factors in premature newborns with hearing disorders. | Latin American Food Security Classifcation (ELCSA) | FI as ordinal variable (4 levels of FI) | Neonatal hearing disorders | Biochemical data, drugs prescribed for premature babies, mother’s age, mother’s age at frst pregnancy, weight gain, weight, length and cephalic perimeter | 81.9% in case group and 54,3% in control group. | Severe food insecurity was a risk factor (OR=12.75, 95%CI = 2.89- 56.16) for hearing disorders, while household food security had a protective effect (OR=0.26, 95%CI 0.08-0.83). |
| Charmichael et al., 2007  (USA) | 1,189 newborns with congenital birth defects (cases) and 695 without congenital birth defects (controls) in California | Case-control study. Investigation of food insecurity as a risk factor for neural tube defects (NTD), orofacial clefts, and conotruncal heart defects | 5 of the 6 questions from the Short form of HFSS | FI as continous varible | Congenital birth defects | Race, education, Prepregnancy BMI, intake of folic acid and energy, stresfullevents | 6- 11% of FI on the cases  3-9% of FI on the controls | Higher scores of FI was associated with increased risk of all of the phenotypes. The adjusted OR(s) were: OR=1.12 spina bifida; OR=0.9 cleft lip. For the association with cleft palate and d-transposition of the great arteries, BMI was an interaction and for tetralogy of Fallot, folic acid intake |
| De Oliveira et al., 2015  (BRAZIL) | 428 brazilian pregnant women from public health network. | Cross-sectional investigative study of socioeconomic variables (including moderate / severe FI), association with anemia | Food Insecurity Brazilian Scale (EBIA) | FI was evaluated considering two levels (food security and mild FI versus moderate and severe FI) | Anemia  (Hb<11g/dl) | Number of family members, age and smoking | Moderate and severe FI – 16,1%  Food security (FS) and Mild FI –83,9% | Moderate FI was  20% in pregnant women with anemia and 14.4% in those without anemia.  Association between FI and anemia was found (p <0.01) |
| Dolatian et al., 2018  (Iran) | 674 pregnant women of Ilam Province (Iran) | Longitudinal study to determine the relationship between psychosocial factors, and food insecurity with preterm delivery. | HFIAS – FANTA - USAID | FI as ordinal variable (4 levels of FI) | Premature delivery | Stress, anxiety, depression, social support, violence, pregnancy's worries. | 34.3% of FI  3.9% of FI and premature birth | The prevalence rates of preterm delivery in cases with FI were 2 times higher than those who had FS. |
| Eaton et al., 2014  (USA and SOUTH AFRICA) | 95 pregnant women who used alcohol  from South Africa | Prospective cohort evaluated through interviews at three moments. Alcohol consumption was investigated in association with FI. | HFSS_USDA with adaptations*** | FI was evaluated considering two levels  (FS versus FI) | Alcohol consumption | Age, ethnicity, education and marital status | 87% of FI | There was significant association between alcohol consumption and FI (p<0.05)  For each item on the scale, more than half of women experienced FI |
| Gamba et al., 2016  (USA) | 688 NHANES’  pregnant women | Cross-sectional study investigating FI  in association with the dietary quality of the pregnant woman | HFSS – USDA | FI was evaluated considering two leves (FS versus FI) | Dietetic quality evaluated by AHEI-P (Alternate Healthy Eating Index modified for Pregnancy) with 1-2 24-hour recalls | Age, place of birth, marital status, ethnicity, education and family income | 19% of FI and 14% on the limite of FS | No significant association between FI and diet quality, but FI gives 2.3 more chance of high calcium intake |
| Garman et al., 2019  (South Africa) | 384 pregnant women living in low income setting in South Africa | Randomised controlled trial with brief psychosocial intervention for perinatal depression to identify trajectories of perinatal depressive symptoms and their predictors, including FI. | HFIAS – FANTA - USAID | FI was evaluated considering two levels (Severe FI or not) | Perinatal depression | No applied, once univariate, rather than multivariate, analyses were preferable given that the objective of the study was to identify high-risk groups more likely to suffer from severe and chronic symptoms, rather than to understand the complex interactions of risk factors. | 29.2% of severe FI | The OR of being classified in the antenatal and postnatal depression were 2.5 times greater on FI women (95%CI: 1.21, 5.15; p-value= 0.013) |
| Gebremedin et al., 2011  ETHIOPIA | 750 pregnant women from Ethiopia | Sectional study to investigate the association between FI, socio-demographic and gestational factors and zinc adequacy | HFIAS – FANTA - USAID | FI as continous variable | Zinc Adequacy/Zinc deficit | Parity, maternal and gestational age | -- | Women with FI scores above the average score had 5 higher risk of zinc deficit |
| Gizaw et al., 2018  ETHIOPIA | 94 cases of low birth weight (LBW) and 376 controls from publics hospitals in Central Ethiopia. | Case-control study to determine the risk factors of LBW, including FI | HFIAS – FANTA - USAID | FI as ordinal variable (4 levels of FI) | Low Birth Weight | Socio-demographic nutritional and reproductive factors. | 53% of FI | FI mothers had about four times higher odds of LBW as compared to FS mothers [AOR = 4.42 (95%CI: 1.02-22.25)] |
| Gross et al., 2018^65^  (USA) | 412 Hispanic / Latino pregnant women from urban New York | Randomized controlled trial analyzing FI effects on prenatal and on feeding practices from birth to10-month-old babies. There was nutritional and breastfeeding counseling intervention | HFSS – USDA | FI was evaluated considering two levels (FS versus FI) categorized as never, prenatal only, infancy only, or both. | Maternal child feeding styles and practices, and depression was also investigated during prenatal period | Schooling, marital status, employment, country of birth, having another child, pre-gestational depression, pre-gestational weight and material difficulties, IF situation of the intervention group | 32% of FI | Women with prolonged food insecurity were more likely to have prenatal depressive symptoms There were no intervention status group differences in FI during either the prenatal or infancy periods. |
| Hanselman et al., 2018  (USA) | 250 Binomials mothers (over 16 years old) and children of Tanzania. | Longitudinal study to explore the risk factors associations, including FI on the dietary practices of children since breastfeeding beginning | HFIAS – FANTA - USAID | FI was evaluated considering two levels (FS versus FI) | Breastfeeding status and feeding practices after birth and up to 6 months of age | Mutual adjusted model | 31,1% of FI | FI was associated with the early introduction of breastfeeding only among women from families who were not landowners with livestock (p-value=0.02) |
| Heyningen et al., 2017  (SOUTH AFRICA) | 376 pregnant women over 18 years old from South Africa | Cross-sectional investigative study of prenatal anxiety in low-income pregnancy in association with socio-demographic and psychosocial factors including FI | HFSSM short version referred to 6 retrospective months | FI was evaluated considering two leves (FS versus FI) | Anxiety and depression related disorders | | Age-adjusted final model, SES level, primigravity, pregnancy trimester, living with a partner, unplanned and unwanted pregnancy, previous aborption | | --- | | 42% of FI | FI women were two and a half times more likely to have a diagnosis of anxiety (OR=2.57; 95%CI% 1.48-4.46). FI was the third factor more related to anxiety. |
| Hoseini et al., 2018  (IRAN) | 860 women after childbirth referred from health centrers in Qazvin province (Iran) | Cross-sectional study to investigate the relationship between FI and pregnancy complications, such as preeclampsia, gestational diabetes and anemia | HFIAS – FANTA - USAID | FI was evaluated considering two levels (FS versus FI) | Pregnancy complications (preeclampsia, gestational diabetes and anemia) | Age, education level, occupation of the mother, level of education, occupation of the spouse, household economic status, number of pregnancies, wanted or unwanted pregnancies, prepregnancy BMI and weight gain during pregnancy. | 32.3% of FI | OR (95%CI) for having a total pregnancy complication 1.64 [1.06–2.54]) in FI group compared to FS. For gestational hypertension OR was 1.24 (95%CI 0.58-2.69), for preeclampsia OR= 3.88 (95%CI 1.18-12.83), for anemia OR=1.24 (95%CI 0.58-2.71) and for gestational diabetes OR=1.63 (95%CI0.81-3.30). |
| Hromi-Fiedler et al., 2011  (USA) | 135 low-income latin pregnant women >  18 years old from Connecticut (USA) | Cross-sectional study investigating the association of FI with depressive illness. | Adapted version from HFSSM.  Adapted in 15 items*** | FI was evaluated considering two levels (FS versus FI) | Depressive symptoms | Socioeconomic and demographic factors, perceived health measures, pre-pregnancy BMI, smoking, alcohol, previous history of depression and marital status | 37% of FI | FI pregnant women were more likely to have depressive symptoms than those without FI. The OR for those with FI to have depression was 2.6 in relation to  the FS ones |
| Jebena et al. 2015  BELGIUM | 642 pregnant women from 11 Ethiopian health centers | Cross-sectional study to evaluate the association of FI with mental stress | HFIAS/FANTA/  USAID | FI was evaluated considering two leves (FSversus FI) | Mental distress | Age, occupation, monthly income, land ownership for agriculture | 9% of moderate FI | The prevalence of mental distress was higher among pregnant women with FI (48.3%) when compared to those who were FS (19.9% ​​p-value<0.05). The FI pregnant women were 4 times more likely to have mental distress then the FS ones |
| Kang et al., 2018  (USA) | 589 pregnant and 641 lactating women (not included in this review) in rural Malawi (Oriental Africa) | Cross-sectional study to examine the association between household food insecurity and dietary diversity | HFIAS/FANTA/  USAID | FI as ordinal variable  (4 levels of FI) | Dietary diversity | Household water sources and sanitation facilities, pregnancy history, education level, occupational status, and health and nutrition service utilization | 66.7% of moderate or severe FI in pregnant women | Severe FI, compared with FS led to a 0.36 lower DDS (p-value<0.05) and the OR=3.19 (95%CI 1.04-13.7) of FI women without meat/fish consuming. To the group without eggs consuming OR=3.77 (95%CI 1.04-13.7) for moderate FI compared to FS. |
| Laraia et al., 2010  (USA) | 810 low income pregnant women in from North Carolina | Longitudinal study with data from the Prospective Pregnancy, Nutrition and Infection cohort to retrospectively assess FI and pregnancy complications. | HFSS/USDA | FI evaluated as an ordinal variable (FS; Marginal FS; FI) | Pregnancy-related complications (pregnancy-induced hypertension, second trimester anemia, and gestational diabetes mellitus) and gestational weight gain. | Age, number of children, income / poverty ratio, physical activity, race, smoking, BMI, education, gestational age as total gestational weight gain estimate | 76% of FS; 24% of marginal FS; 10% of FI | FI was associated with pre-gestational obesity, high gestational weight gain, and high adequacy of weight gain ratio in the adjusted analysis. Marginal FS was associated with gestational diabetes in the adjusted model |
| Laraia et al., 2013  (USA) | 1041 pregnant women > 16 years with pré-pregnancy dietary restriction, from North Carolina | Longitudinal study with data from the Prospective Pregnancy, Nutrition and Infection cohort to retrospectively assess FI  and high gestational weight gain in women | HFSS/USDA | FI evaluated as an ordinal variable (FS; Marginal FS; FI) | Gestational weight gain and adequacy of gestational weight gain based on IOM recommendations | Age, education, race, income / poverty ratio, marital status, number of children, smoking in the first 6 months, physical activity, pre-gestational BMI and gestational weeks | 12,3% of marginal FS | Marginal FS and low pre-pregnancy dietary restriction were associated with lower weight gain. Marginal FS and high dietary restriction were associated with high gestational weight adequacy and gain. |
| Laraia et al., 2015  (USA) | 688 pregnant women from North Carolina cohort | Longitudinal study to assess the influence of pregnancy and postpartum FI on stress, fat intake, weight and eating disorders | HFSS/USDA | FI evaluated as anordinal variable (FS; Marginal FS; FI) | Stress, eating disorders, fat consumption, body mass in pregnancy and postpartum | Sociodemographic and economic characteristics and health behaviors  (physical activity and smoking) breastfeeding and poverty level | Pregnancy: 8% of marginal FS; 5,2% of FI  Post partum: 4,4% of marginal FS; 6% of FI | FI level and pre-gestational weight were interacted. Stress and poor eating attitudes were higher for marginal FS and FI pregnant women than for FS ones |
| Lebso et al., 2017  ETHIOPIA | 507 pregnant women from Southeast Ethiopia | Cross-sectional study to verify factors associated with anemia in pregnancy, including FI | Adapted from HFIAS for the local context*** | FI as ordinal variable (4 levels of FI) | Anemia in pregnancy | Household income, FI level, toilet availability, frequency of meals per day, consumption of animal sources at least once / week,  history of malaria and nutritional status | 21,4% of FS; 17,9% of mild FI; 41,7% of moderated FI; 19% of severe FI | No FI levels were significantly associated with anemia in pregnancy. |
| Miller, 2017  (EUA) | 266 HIV + pregnant women from Puerto Rico and USA | Longitudinal study to verify the quality of the diet predicted by the socio-demographic and gestational covariates (including FS). The association between diet quality and low birth weight and gestational age was verified. | Short version of HFSSM | FI was evaluated considering two leves (FS versus FI) | Birth weight and gestational age in infants of HIV infected women.  For FI, diet quality is an outcome | FI was considerd a covariate variable in the relationship between diet quality and birth weight and gestational age in infants of HIV infected women | 62% of FI | FI was not one of the factors associated with the diet’s quality |
| Moafi et al., 2018  (IRAN) | 394 pregnant women in Qazvin city, Iran. | Cross-sectional study to investigate the effect of food insecurity on quality of life. | HFIAS/FANTA/  USAID | FI as ordinal variable (4 levels of FI) | Quality of life | Age, education level and job, husband’s education level and job, family living place, living house ownership status, perceived economic status, current gestational rank, gestational age, number of children, pregnancy willingness status and gender of fetus | 43.9% of FI | FI women had the lowest score due to physical reasons domain of quality of life (68.6±40.4, 61.3±45.5 & 51.3±47.7) respectively for mild, moderate and severe FI. One unit reduction of FI decreased the total quality of life score by 5.2 score (95%CI -9.7-−0.7) among the mild FI group, 10.8 score (95%CI -17.1-−4.6) among the moderate FI group and 14.1 score (95%CI -19.7-−8.5) among the severe FI group. |
| Murray et al. 2015  (AUSTRA-LIA) | 431 pregnant women from Vietnan central region | Cross-sectional study to investigate the prevalence of postpartum depression and its socio-cultural factors including FI | Considered one question about the numbers of months during the past year whose women could not afford to buy the food their family needed | Categorical variable considering the numbers of months families could not afford to buy food  (every months, every 1-6 months, every 6-12 months, never) | Postnatal mood disturbance  (depression and wellbeing) | Age, education,  urban or rural area, socioeconomic class, previous births | 39% of  Pregnant women without  money to buy food every month | FI reporting lack of resources throughout the month was significantly associated with the depression score (β = 2.27; p <0.01) and lack of recourse every 1 to 6 months (β = 1,655; p <0.01).  FI was significantly associated with well-being. |
| Na et al., 2016  (USA) | 14.600 pregnant women from Bangladesh rural zone | Longitudinal associations as a part of a cluster-randomized trial of prenatal multiple micronutrient  supplementation | HFIAS/FANTA  USAID adapted in recall time (at 6 mo postpartum)*** | FI as ordinal variable  (4 levelsof FI) | Maternal dietary diversity score (DDS) | Number of “visits”, seasonality, maternal age, arm circumference at baseline, number of rooms, religion, education and income | 49.7% of FI  (mild FI=20.1%; moderated FI=15.4%; severe FI=14.2% | The DDS decreased with progressively worse FI, compared with women from food secure households. |
| Natamba et al., 2017  (USA) | 403 HIV‐infected and HIV uninfected pregnant women  from northern Uganda | Cross-sectional study evaluated dutring a longitudinal observational study to investigate de relationship between FI and prenatal depression | Nine item individually  FI scale (IFIAS) to assess perceived FI in the past 4weeks | FI as continous score variable (0 until 9) | Prenatal depressive symptoms | Participants age in  years, HIV status, parity, marital status, education level, domestic violence and former stay in a camp | -- | FI was associated with depressive symptoms severity moderated by Social Support (SS). FI was stronger among women in the low SS category than for women belonging to the high SS group |
| Nunnery et al., 2017  (USA) | 198 low-income pregnant women in a Southeast region of the USA | Cross sectional survey to determine differences in the availability of variety of fruits and vegetables by food security status. | HFSS/USDA  Adapted for pregnancy condition**** | FI as ordinal variable  (4 levels of FI) | Availability of variety of fruits (F) and vegetables (V) | Age, monthly household income, education, race/ethnicity, household size and participation in a Supplemental Nutrition Assistance Program (SNAP). | 43% of low FS (LFS)/very low FS (VLFS) | VLFS led to a lower variety of fruits (p-value=0.028) and vegetables (p-value=0.058). FS was associated with the daily intake of F&V (indirect effect (95%CI: fresh fruits= −0·039 (−0·074, −0·013); fresh vegetables, −0·048 (−0·083, −0·023). As FS worsened, variety of F&V decreased. |
| Onah et al., 2016  (South Africa) | 376 pregnant women in Cape Town  (South Africa) | Cross-sectional study to determine the association between risk factors such as demographic, economic, and psychosocial factors as predictors of alcohol and drug use. | Short version of HFSSM | FI as ordinal variable  (3 levels of FI: food secure, food insecure, food insufcient) | Alcohol or drug (AOD) use | Age, language, education, marital status, socioeconomic status (SES), obstetric information, whether the pregnancy was planned, wanted, as well as past psychiatric history | 42% of FI and 12% of food insufficiency | Women that were food insecure were more likely to use AODs (OR=1.04, 95%CI 0.42–2.59), however, women that were food insufcient were three times more likely to use AODs (OR= 3.73, 95%CI 2.32–4.29) |
| Rose-Jacobs et al., 2018  (USA) | 75 pregnant women; with opioid agonist treatment at Boston Medical Center in Boston, (2013–15) | Cross-secrional study, part of a prospective cohort to estimate the association between prenatal FI and neonatal abstinence syndrome (NAS) severity. | A validated two-question Hunger Vital Sign™ based on the US HFSS***** | FI was evaluated considering two leves (FS versus FI) | Neonatal abstinence syndrome | Past-week maternal depressive symptoms, maternal opioid agonist at time of birth; tobacco smoking, illicit drug use, psychiatric medications; and breastfeeding initiation | 57.3% of FI | FI women were more likely to have infants requiring any NAS treatment in analyses controlled for prenatal: maternal depression (AOR = 3.69, 95%CI 1.02-13.43) or adjusted by the use of methadone opioid agonist (AOR= 4.17, 95% CI = 1.05, 16.50). Smoking or breastfeeding did not modified the association of FI and pharmacological NAS treatment |
| Sidebottom et al., 2013  (USA) | 594 pregnant women from Minneapolis and St. Paul Health Centers in Minnesota. | Longitudinal study to investigate predictors of prenatal and postpartum depression | HFFS adapted with 4 items*** | FI was  Evaluated Considering two leves (mild FI versus moderated+severe FI) | Postpartum Depressive Symptom | Social, behavioral and environmental risk factors | 58,7% of mild FI; 41,3% of moderated+severe FI | After adjustment, moderate/severe FI was associated with elevated depressive symptom levels only in the prenatal periaod |
| Tsai et al., 2016  (USA/SOUTH AFRICA) | 1,238 pregnant women from South Africa | Cross-sectional study in  cluster-randomized trial | Food insufficiency as a proxy for FI. A only question was considered (“How many days in the past week have you gone hungry? By this I mean days when you felt you didn’t have enough to eat.”) | FI as continous varible | Quartiles of depression symptom severity | Age, employement, father of child presence, HIV serostatus, alcohool intake, monthly household income, self reported diseases (diabetes and hypertension) | -- | Food insufficiency had significant association with depression among women with low levels of instrumental support. the adverse impacts of Severe food insufficiency were experienced by women with severe depression symptons |
| [Webb-Girard](https://www.ncbi.nlm.nih.gov/pubmed/?term=Webb-Girard A%5BAuthor%5D&cauthor=true&cauthor_uid=20874844) et al., 2012  USA/  KENYA/  CANADA | 148 women who were either pregnant or with a child ≤24 months, Nakuru, Kenya | Cross-sectional quantitative and qualitative study to investigate the FI women’s beliefs and attitudes towards exclusive breastfeeding | HFIAS – FANTA – USAID/HHS | FI was evaluated considering two leves  (FS/ mild FI versus moderated/severe  FI) | Beliefs and attitudes towards  exclusive breastfeeding | Maternal age, parity,  years of study,  state of pregnancy; contribution to family income;  household size; infant feeding counseling | 22% of FS/Mild FI;  78% of Moderate FI/SevereFI | Pregnant women with moderate+severe FI had the OR=2.6 higher in believing in the maintenance of breastfeeding for 6 months than those with FS+Mild FI |
| Widen et al., 2018  (USA) | 333 pregnant and lactating HIV+ women in Kenya receiving antiretroviral therapy | Longitudinal study carried out between 2014 and 2016, to evaluate the role of HIV infection and FI on body composition during pregnancy and lactation. | Individual Food Insecurity Access Scale (IFIAS) | FI as continous varible | Adverse body composition changes | Height, age, education parity, depression, fever gastrointestinal distress,dietary diversity, hunger season in the previous month and proportion of heavy work | -- | FI was inversely associated with Arm Muscular Area (AMA) and Mid-upper Arm Circumference (MUAC) postpartum (AMA ß-range = -0.47 to -0.92 cm^3^; MUAC ß-range= -0.09 to -0.15 cm; all p-value <0.05). |
| Woldetensay et al., 2018  (Germany) | 4680 pregnant women between 12 to 32 weeks of gestation in Ethiopia | Longitudinal study, carried out from 2014 to 2016 to investigate the association between nutritional and socio-demographic factors, such as FI and prenatal depression. | HFIAS/  FANTA – USAID/HHS | FI as ordinal variable  (4 levelsof FI) | Prenatal depressive syndrome | Age, religion, marital status, family size and wealth index, obstetric factors, acute illnesses, social support, chat chewing practices and intimate partner violence | 87.2% of FI  39.4% of moderate FI  13.5% of severe FI | Adjusting for confounding variables, moderate household FI (OR=1.74; 95%CI 1.31-2.32), severe household FI (OR=7.90; 95%CI: 5.87-10.62) were significantly associated with prenatal depressive symptoms. |

(i) ‘How often do you not have enough money to afford the kind of food you and your family should have?, adapted from an economic strain measure; (ii) ‘In the past 6 months, did you go without food because you did not have the money to pay for it?’from the Crisis in Family Systems-Revised measure. Adapted from:

*

Shalowitz MU, Berry CA, Rasinski KA, et al., 1988.

** Adapted from Melgar-Quinonez et al., 2006.

*** Adapted instruments

**** Adapted as follow: the standard 30d or 1-year reference period used for the survey statements was changed to ‘Since you’ve become pregnant or in the past few

months…’.

***** Adapted instrument categorizing pregnant women as FI if one or more screening questions were endorsed

# AOR = Adjusted odds ratio

# 95% CI = 95% Confidence interval
