# Supplementary material for: Household food insecurity associated with gestacional and neonatal outcomes: a systematic review
Source: BMC Pregnancy Childbirth. 2020 Apr 17;20:229. doi: 10.1186/s12884-020-02917-9 (PMC7164154; doi:10.1186/s12884-020-02917-9)
Supplement: Supplementary file 2 — Additional file 2:Table S2. Proportion (%) and number (n) of studies of clinical or nutritional outcomes observed in pregnant women and newborns associated with food insecurity, identified in the present review Table referent to the proportions of outcomes investigated in this systematic review. [file 12884_2020_2917_MOESM2_ESM.docx]

Table 2: Proportion (%) and number (n) of studies of clinical or nutritional outcomes observed in pregnant women and newborns associated with food insecurity, identified in the present review.

| **Clinical or nutritional adverse outcomes** | **Proportion of total studies/(n)** |
| --- | --- |
| ***Gestational outcomes*** | **81% (30)** |
| Depression / Stress / Anxiety | 29.7% (11) |
| Consumption/Dietary quality/Dietary diversity | 22.4% (8) |
| Weight gain / nutritional status of the pregnant women (*) | 5.4% (2) |
| Diverse clinical complications (diabetes, hypertension and others) | 5.4% (2) |
| Anemia(**) | 5.4% (2) |
| Alcohol and/ or drug consumption | 5.4% (2) |
| Antiretroviral drugs pharmacocinetic | 2.7% (1) |
| Quality of life | 2.7% (1) |
| Pregnant women beliefs toward breastfeeding | 2.7% (1) |
| ***Neonatal outcomes*** | **19% (7)** |
| Mortality | 2.7% (1) |
| Low Birth Weight | 2.7% (1) |
| Breastfeeding interruption | 2.7% (1) |
| Prematurity | 2.7% (1) |
| Birth congenital defects | 2.7% (1) |
| Hearing disorders | 2.7% (1) |
| Neonatal abstinence syndrome | 2.7% (1) |

(*) Outcome related to weight gain and nutritional status exclusively

(**) Outcome related to anemia exclusively
