# Supplementary material for: Household food insecurity associated with gestacional and neonatal outcomes: a systematic review
Source: BMC Pregnancy Childbirth. 2020 Apr 17;20:229. doi: 10.1186/s12884-020-02917-9 (PMC7164154; doi:10.1186/s12884-020-02917-9)
Supplement: Supplementary file 3 — Additional file 3. Quality Assessment. The Joanna Briggs Institute Reviewers’ Manual 2015 of quality assessment. Instrument from the Joanna Briggs Institute to assess the quality of the studies included in this systematic review. [file 12884_2020_2917_MOESM3_ESM.docx]

| **Author** | **Year** |  |  | **Joanna Briggs Assessment of studies quality** | | | | |  |  |  |
| --- | --- | --- | --- | --- | --- | --- | --- | --- | --- | --- | --- |
| Ayyub et al. | 2018 | 1 | 2 | 3 | 4 | 5 | 6 | 7 | 8 | 9 | **Conclusion** |
| Bartelink et al. | 2014 |  |  |  |  |  |  |  |  |  | Inclusion |
| Borders, et al. | 2015 |  |  |  |  |  |  |  |  |  | Inclusion |
| Brunst et al. | 2014 |  |  |  |  |  |  |  |  |  | Exclusion |
| Campbell et al. | 2009 |  |  |  |  |  |  |  |  |  | Inclusion |
| Carmichael et al. | 2007 |  |  |  |  |  |  |  |  |  | Inclusion |
| Castillo Chávez et al. | 2019 |  |  |  |  |  |  |  |  |  | Inclusion |
| Cheu et al. | 2019 |  |  |  |  |  |  |  |  |  | Exclusion |
| Chomat et al. | 2015 |  |  |  |  |  |  |  |  |  | Exclusion |
| [De Oliveira](javascript:searchAuthor('De%20Oliveira,%20A.%20C.')) et al. | 2015 |  |  |  |  |  |  |  |  |  | Inclusion |
| Dolation et al. | 2018 |  |  |  |  |  |  |  |  |  | Inclusion |
| Eaton et al. | 2014 |  |  |  |  |  |  |  |  |  | Inclusion |
| Gamba et al. | 2016 |  |  |  |  |  |  |  |  |  | Inclusion |
| Garman et al. | 2019 |  |  |  |  |  |  |  |  |  | Inclusion |
| Gebremedhin et al. | 2011 |  |  |  |  |  |  |  |  |  | Inclusion |
| Gizaw et al. | 2018 |  |  |  |  |  |  |  |  |  | Inclusion |
| Gross et al. | 2018 |  |  |  |  |  |  |  |  |  | Inclusion |
| Hanselman et al. | 2018 |  |  |  |  |  |  |  |  |  | Inclusion |
| Heyningen et al | 2016 |  |  |  |  |  |  |  |  |  | Inclusion |
| Hoseini et al. | 2018 |  |  |  |  |  |  |  |  |  | Inclusion |
| Hromi-Fiedler et al. | 2011 |  |  |  |  |  |  |  |  |  | Inclusion |
| Jebena et al. | 2015 |  |  |  |  |  |  |  |  |  | Inclusion |
| Kang et al. | 2018 |  |  |  |  |  |  |  |  |  | Inclusion |
| Laraia et al. | 2010 |  |  |  |  |  |  |  |  |  | Inclusion |
| Laraia et al. | 2013 |  |  |  |  |  |  |  |  |  | Inclusion |
| Laraia et al. | 2015 |  |  |  |  |  |  |  |  |  | Inclusion |
| Lebso et al. | 2017 |  |  |  |  |  |  |  |  |  | Inclusion |
| Miller et al. | 2017 |  |  |  |  |  |  |  |  |  | Inclusion |
| Moafi et al. | 2018 |  |  |  |  |  |  |  |  |  | Inclusion |
| Morales et al. | 2016 |  |  |  |  |  |  |  |  |  | Exclusion |
| Murray et al. | 2015 |  |  |  |  |  |  |  |  |  | Inclusion |
| Na et al. | 2016 |  |  |  |  |  |  |  |  |  | Inclusion |
| Natamba et al. | 2017 |  |  |  |  |  |  |  |  |  | Inclusion |
| Nunnery et al. | 2017 |  |  |  |  |  |  |  |  |  | Inclusion |
| Onah et al. | 2016 |  |  |  |  |  |  |  |  |  | Inclusion |
| Park et al. | 2014 |  |  |  |  |  |  |  |  |  | Exclusion |
| Quintero Tabares et al. | 2010 |  |  |  |  |  |  |  |  |  | Exclusion |
| Restrepo-Mesa et al. | 2014 |  |  |  |  |  |  |  |  |  | Exclusion |
| Restrepo-Mesa et al. | 2015 |  |  |  |  |  |  |  |  |  | Exclusion |
| Rose-Jacobs et al. | 2018 |  |  |  |  |  |  |  |  |  | Inclusion |
| Saaka et al. | 2017 |  |  |  |  |  |  |  |  |  | Exclusion |
| Saeed et al. | 2017 |  |  |  |  |  |  |  |  |  | Exclusion |
| Sidebottom, A. C. | 2013 |  |  |  |  |  |  |  |  |  | Inclusion |
| Tsai, A. C. | 2016 |  |  |  |  |  |  |  |  |  | Inclusion |
| [Webb-Girard, A.](javascript:searchAuthor('Webb-Girard,%20A.')) | 2012 |  |  |  |  |  |  |  |  |  | Inclusion |
| Widen et al., 2016 | 2016 |  |  |  |  |  |  |  |  |  | Inclusion |
| Woldetensay et al., 2018 | 2018 |  |  |  |  |  |  |  |  |  | Inclusion |

|  | does not meet the criteria | |  |
| --- | --- | --- | --- |
|  | meet the criteria |  |  |
|  | Not applicable |  | |
